# Aiming accuracy in preferred and non-preferred limbs: implications for programing models of motor control

**DOI:** 10.3389/fpsyg.2014.01236

**Published:** 2014-11-04

**Authors:** David E. Sherwood

**Affiliations:** Motor Behavior Laboratory, Department of Integrative Physiology, University of Colorado at Boulder, CO, USA

**Keywords:** motor equivalence, motor programing, spatial accuracy, dominant and non-dominant limbs, temporal structure

## Abstract

Most models of motor programing contend that one can perform learned actions with different muscle groups or limbs demonstrating the concept of motor equivalence. The goal of this review is to determine the generality of this concept within the context of aiming movements performed by both preferred and non-preferred limbs. Theoretical approaches to motor programing are described, followed by a comparison of a variety of kinematic measures taken from preferred and non-preferred limbs from simple and more complex aiming tasks. In general, the support for motor equivalency is strong for one- and two-dimensional aiming tasks and for simultaneous bimanual movements, but mixed for unconstrained throwing tasks and tasks that require feedback-based corrections.

One of the more persistent concepts of human motor control has been that of motor equivalence. The idea that one can achieve the same goal with different muscle groups or limbs has been proposed and described by several prominent researchers since the early 1900s (Head, 1920; Bartlett, 1932; Bernstein, 1967; Schmidt, 1975). Common examples include the ability of basketball players to shoot and dribble with either hand with equal proficiency, and for marked bilateral transfer in handwriting. At the same time there appear to be significant bilateral control differences between preferred and non-preferred limbs for a variety of motor tasks (e.g., Carson, 1989). Therefore, the goal of the present paper is to explore the concept of motor equivalency by investigating the differences between preferred and non-preferred limbs within the context of throwing and aiming movements in keeping with the topic on manual asymmetries, handedness, and motor performance. Theoretical approaches are described first, followed by sections describing the kinematic differences between preferred and non-preferred upper limbs in a variety of contexts (aiming movements and throwing) and experimental paradigms (adaptation studies and bimanual movements).

## THEORETICAL APPROACHES

Bernstein (1967, p. 49) captured the idea of motor equivalence perfectly in the following quote:

It is clear that each of the variations of a movement (for example, drawing a circle large or small, directly in front of oneself or to one side, on a horizontal piece of paper or on a vertical blackboard) demands a quite different muscular formula; and even more that this, involves a completely different set of muscles in the action. The almost equal facility and accuracy with which all these variations can be performed is evidence for the fact that they are ultimately determined by one and the same directional engram in relation to which dimensions and position play a secondary role.

For Bernstein, the engram was a central nervous system structure responsible for the control of both spatial and temporal movement characteristics. The engram controlled the entire movement, especially the order of the muscular contractions and the overall rhythm. These central features of the movement, which remained relatively constant from trial to trial, were termed topological characteristics and were controlled by the highest level of a hierarchical control system. At a lower level of the system, metrical characteristics (i.e., magnitude, muscle group) allowed for variations in expression of the engram and motor equivalency.

Schmidt (1975) made Bernstein’s notions about the structure of the engram more explicit in the context of schema theory. According to the theory, motor equivalency was a result of the formation of a generalized motor program (GMP). The GMP was defined by so-called invariant characteristics that remained constant from performance to performance, but were different for different classes of movement (i.e., throwing, kicking). Schmidt (1975) identified relative timing, the sequence of events, and relative force as the invariant features of the GMP. Relative timing is defined as the proportion of the total time required by any phase of the movement (e.g., the proportion of total time taken by the stance phase in gait) and was thought to be invariant across changes in the overall movement time. Relative force is defined by the ratio of agonist/antagonist muscle activity, or by the relative magnitudes of flexion and extension movements, for example. The GMP is a flexible control structure because variable parameters could be used to change the movement outcome without requiring the use of a unique program. Overall duration, force, and muscle group were considered parameters that could all be varied across trials to change to outcome of the GMP. Therefore, the parameters are varied to change the magnitude of the movement from smaller to larger in the case of handwriting, or change the limb with which an object is thrown by changing the muscle group involved.

In the case of bilateral transfer, both the Bernstein’s (1967) and Schmidt’s (1975) ideas predict positive transfer because the same engram or GMP is used to control either limb. The invariant features would be preserved for use in both cases, while the parameters could be varied individually for each limb. However, the presence of manual asymmetries in the performance of the preferred and non-preferred limbs has required theoretical approaches involving the unique contribution of each cerebral hemisphere in the motor control process (Pan and van Gemmert, 2013).

For example, Hicks (1974) and Taylor and Heilman (1980) showed asymmetrical transfer such that the right hand benefited more than the left hand from opposite hand training. They proposed what has been referred to as an access model (Parlow and Kinsbourne, 1989) that states that a single motor program was stored in the left (dominant) hemisphere as a result of practice with either the preferred or non-preferred limb. The right hand benefits more than the left hand because the right hand has direct access to the information in the left hemisphere. The left hand has only indirect access to the information in the left hemisphere via the corpus callosum. The main limitations of the access model are that bilateral transfer is unidirectional and it cannot explain the results of studies showing that the left hand benefits more than the right from opposite hand training (e.g., Ammons and Ammons, 1951). As an alternative to the access model, Parlow and Kinsbourne (1989) proposed the so-called cross-activation model. In this case practice with the preferred limb creates motor programs in both the dominant and non-dominant hemispheres, although the program is weaker in the non-dominant hemisphere. Training only the non-dominant hemisphere only creates a motor program in the non-dominant hemisphere. According to this model bilateral transfer is always stronger from the preferred limb to the non-preferred limb than vice verse due to the lack of a motor program in the dominant hemisphere after non-preferred limb practice. As with the access model, only transfer from the preferred to the non-preferred limb can be explained. More recently Sainburg and colleagues (Sainburg and Wang, 2002; Wang and Sainburg, 2004, 2006) have argued that the preferred and non-preferred hands have access to all the information learned by the opposite hand, but controllers unique to each hand select and use information differently. For example, the dominant hemisphere mechanisms underlie specification of the shape and direction of the movement trajectory, while the non-dominant hemisphere specializes in final limb position.

## MOTOR EQUIVALENCY IN AIMING MOVEMENTS

One way to organize the vast amount of information on aiming movements is to first describe the differences between preferred and non-preferred limbs in the simplest tasks involving one- and two-dimensional movements, followed by work on unconstrained three-dimensional movements. If the concept of motor equivalency is truly a general one, then evidence should be available for all aiming tasks.

Perhaps the simplest aiming movement studied involves moving a lever or joystick so a cursor reaches a target displayed on a computer screen. Spatial errors can only be made in the single dimension of distance, and movement time can be controlled with instructions and augmented feedback. On such study was performed by Zuoza et al. (2009) in right-handed male participants, where the goal was to move a joystick “quickly and accurately” so the cursor reached the target in 400–600 ms without concurrent visual feedback. The preferred limb was more accurate than the non-preferred limb, although when errors were made the preferred limb tended to undershoot the target and the non-preferred limb tended to overshoot the target. In absolute measures, the non-preferred limb spent less time in deceleration than the preferred limb and showed greater peak and average velocity than the preferred limb as well. However, such differences could simply be attributed to changes in parameters of the GMP as discussed earlier. The preferred limb spent 42 and 58% of the total time in the acceleration and deceleration phases, respectively, compared with 45 and 55% for the non-preferred limb, suggesting a very similar relative timing pattern for both limbs. In another study, participants moved a lever in the horizontal plane different distances (5^°^, 10^°^, 20^°^, 30^°^, and 40^°^) with each arm at a self-selected speed (Al-Senawi and Cooke, 1985). There was no difference in spatial error between the limbs and the velocity profiles were nearly identical as well. These results support the GMP explanation for motor equivalency since it is likely the same motor program was used for the control of both limbs. In addition, the Al-Senawi and Cooke (1985) study supports Bernstein’s notion about the facility of transfer between the limbs.

Two-dimensional aiming tasks are typically done on digitizing tablets or other surfaces using a computer mouse where the target is displayed on the testing surface or on the computer screen. Errors can be made in the horizontal and/or vertical planes and concurrent visual feedback can be provided or limited. In one study, Sainburg and Kalakanis (2000) participants moved to targets requiring 20^°^ of elbow excursion and either 5^°^, 10^°^, or 15^°^ of shoulder excursion without concurrent visual feedback with both preferred and non-preferred limbs. Target accuracy, and elbow and shoulder joint angles were computed for both limbs. Unlike one-dimensional aiming tasks, the hand paths were highly curvilinear with the left hand showing a “left to right” path and the right hand a “right to left” path, but target accuracy was the same in both limbs. Although relative timing was not computed, the ratio of shoulder to elbow excursion was computed for each target condition. The shoulder/elbow ratio taken at the peak velocity was greater for the right hand compared to the left hand, but no difference in the ratio was detected at the final target position. An analysis of the joint torques indicated that each limb to achieve the target used different strategies. The right arm used elbow and shoulder torques synergistically to move the upper arm while the same torques countered one another in the left arm. Overall, the evidence here suggests that different GMPs were used to control the two limbs, although accuracy was equal for both limbs.

The two studies reviewed thus far showed mixed results in terms of motor equivalency when movements were made without the benefit of concurrent visual feedback. Would motor equivalency be shown if visual feedback was available for both limbs? Carson et al. (1993) studied the accuracy and kinematic pattern of aiming movements with various levels of concurrent visual feedback available. Participants had full vision of the arm and target, or just the target, or just the arm, or neither the arm nor target in different conditions. Instructions were also given to be either fast or accurate. The right-handed participants were more accurate with the right hand compared with their left hand across all visual conditions. Although relative timing was not presented, the percentage of time before and after peak velocity could be calculated from the data presented. Under the fast movement instruction 30 and 70% of the total time were spent before and after peak velocity, respectively. Somewhat greater relative time was spent after peak velocity (73.6%) when accuracy was emphasized. However, the relative timing pattern in the left and right limbs was nearly identical across all visual feedback conditions suggesting that the same GMP was used to control both limbs. This finding was supported by Bryden (2002) in a study using Fitts’ paradigm where the index of difficulty (ID) varied from 3.06 to 14.29 bits of information. As predicted by Fitts’ law, movement time increased directly with ID for both the left and right arms. However, there was no difference between the arms on any kinematic measure of performance including the relative time spent in acceleration or deceleration, again supporting the notion of motor equivalency.

Perhaps the most effective test of the concept of motor equivalency is when a metrical characteristic like distance or time is varied and the relative timing structure is compared across limbs. Poston et al. (2009) varied the required angle of aiming movements (either 5^°^, 45^°^, or 85^°^) to the left and right of the participant’s midline. Movements were made until the target was contacted, so spatial errors were essentially 0. Analysis of the kinematics revealed that the movement time, average velocity, the relative length and duration of the primary submovement, and the normalized jerk were nearly identical in both limbs. These results suggest that the same GMP was used for both limbs due to the very similar relative timing patterns in both limbs. Sainburg and Schaefer (2004) varied movement distance (10^°^, 20^°^, 35^°^, and 45^°^) requiring “uncorrected” elbow extensions in both limbs. There were no interlimb differences in spatial accuracy, but somewhat different kinematic patterns were shown between the limbs. In both limbs, peak velocity and the time to peak velocity scaled directly with distance, although the slope of the time to peak velocity/distance relation was greater for the non-preferred limb. Also, the acceleration-time patterns were different. In the non-preferred limb, the initial peak in acceleration was nearly constant across distances, with additional positive peaks emerging during the movement. The initial peak in acceleration scaled directly with distance in the preferred limb. These results suggest that different motor programs were used whereby distance was varied in the non-preferred limb by changing the duration of the acceleration-time pulse, while the preferred limb varied the amplitude of the acceleration-time pulse (cf., Brown and Cooke, 1984; Ghez and Gordon, 1987). It could be that the strategy used by the preferred limb was due to extensive practice dedicated to that limb, and the strategy used by the non-preferred limb could be indicative of relatively novice performance. Roy et al. (1994) instructed participants to make either “fast” or “accurate” movements to targets and showed a very similar relative timing pattern in the left and right limbs in both fast and accurate instructional conditions. However, much less relative time was devoted to the time after peak velocity (55%) in the speed condition compared with the accurate condition (72%), suggesting that a different GMP was used in the two conditions.

One advantage of using two-dimensional aiming movements to investigate the concept of motor equivalence is that the relative timing pattern could be easily determined because the participant decelerates the limb when approaching the target. The case is different when evaluating the relative timing pattern in unconstrained three-dimensional aiming movements, because the pattern of deceleration could be disrupted by the impact of the limb with the target surface. This problem was highlighted by Todor and Cisneros (1985) who investigated the accuracy of aiming movements using a “dart-throwing” motion over 40.64 cm to targets 0.635, 1.27, or 2.54 cm in diameter. Four phases of the acceleration-time trace were identified: T1, time to peak positive acceleration, T2, time from peak positive acceleration to zero acceleration, T3, time from zero acceleration to peak deceleration, and T4, time from peak deceleration to target contact. Trials were classified based on the duration of T4. Trials were labeled “late” if T4 was less than 50 ms, or “early” if T4 was greater than 50 ms. The average T4 duration of the late trials was less than 5 ms, indicating that peak deceleration occurred immediately before target impact. The duration of T4 in the early trials was greater than 100 ms, indicating that the participants were able to slow the limb down to some extent before target impact. Focusing only on the early trials, the relative timing pattern involving T1, T2, and T3 were very similar for the left and right hands suggesting initial use of the same GMP. However, the left hand spent 4% more relative time in T4 on average compared with the right hand, suggesting the left hand required more time to make movement adjustments when approaching the target. Haaland and Harrington (1989) replicated these results by showing similar relative timing for the left hand and right hand for the initial (LH = 53.5%, RH = 51.2%) and corrective (LH = 46.4%, RH = 48.7%) movement phases using the Fitts’ paradigm. Further support for motor equivalency was provided by Barral and Debû (2004) similar proportions of time in the decelerative phase for both limbs for three different target locations in women. In men, the relative timing was the same for two of the three target locations.

## MOTOR EQUIVALENCE IN THROWING

There was clear evidence for motor equivalence in laboratory-based aiming tasks performed with both left and right limbs. The relative timing pattern based on velocity or acceleration-time records was very similar for both limbs in most circumstances. This section reviews studies comparing the preferred and non-preferred limbs in throwing, arguably the least constrained aiming task possible. McDonald et al. (1989) investigated kinematic differences between left and right limbs in dart-throwing in well-practiced participants (500 practice trials for the preferred hand, 1250 for the non-preferred hand). Wrist, elbow, and shoulder joint angles were calculated for the first and last 10 throws for each limb. Accuracy was better in the preferred limb compared with the non-preferred limb, although accuracy improved in both limbs over the practice trials. Within-limb correlations between joints for angular displacement and angular velocity tended to be higher for the non-preferred limb compared with the preferred limb. However, there were strong correlations between the preferred and non-preferred limbs for the resultant displacement and the resultant velocity (all above 0.83) suggesting the same kinematic pattern was shown by both limbs. However, the relative timing pattern was not described in the study nor were data available to calculate the appropriate percentages, so the degree of motor equivalence could not be determined.

Along the same lines, Hore et al. (1996) provided a kinematic analysis of preferred and non-preferred limbs is a seated throwing task. The timing and velocity of proximal joints (shoulder, elbow, and wrist) and distal joints (fingers) were measured and correlated with target accuracy. Target accuracy was better in the preferred limb than the non-preferred limb, and joint rotations were more variable in the non-preferred limb compared to the preferred limb. However, the hand path trajectories were very similar between the left and right arms for a given participant. Different participants showed quite varied “styles” of throwing. Again, the relative timing pattern for each limb was not provided. In a more recent study, Hore et al. (2005), participants threw baseballs at a target at three different speeds with both preferred and non-preferred limbs. Throws with the preferred limb were different from the non-preferred limb in several respects. For preferred limb throws, the joint positions at ball release were different across speeds for the elbow, wrist and shoulder. In addition, participants varied the coordination between joints to achieve throws of different speed. The evidence for a similar relative timing pattern was mixed. There was no evidence for consistent relative timing of elbow, wrist, and shoulder positions across speeds, but there was some evidence for a similar relative timing pattern of the vertical component of the hand path across throwing speeds, but only for the preferred limb. Throws with the non-preferred limb showed relatively small differences in joint motions across speeds compared with the preferred limb. For example, there were no differences in wrist, elbow, or shoulder position at ball release across speed. Further, there was little evidence for a consistent relative timing in the non-preferred limb.

The study by Hore et al. (2005) has some interesting implications for the concept of motor equivalency. Skilled throwers apparently vary the joint coordination pattern in order to change ball speed to accomplish changes in velocity for the preferred limb. However, when learning to throw with the non-preferred limb, they initially use a very similar spatial pattern suggesting they use the same GMP across changes in speed. Also, in a complex coordination task like throwing, the evidence for a consistent relative timing is mixed, and depends on what movement characteristic is evaluated and the throwing limb. The relative timing of some aspects of the hand path and finger opening (Hore and Watts, 2005) are maintained across speeds, but not for elbow, wrist, and shoulder positions.

## ADAPTATION STUDIES

Another experimental design that could be used to evaluate motor equivalency is a design where practice is first provided for either the preferred or non-preferred limb under normal target conditions, and then the same or opposite limb is tested when the target is displaced or visual feedback is rotated, for example. In the study by Sainburg and Wang (2002), participants moved to one of eight targets with goal movement times between 400 and 600 ms, beginning with either the left or right limb. After baseline trials under normal visual feedback conditions, the cursor was rotated 30^°^ relative to the start position and practice continued with the opposite limb. The amount of bilateral transfer depended on what kind of error was evaluated. For direction error at peak velocity, the right arm benefited from left arm training, but the left arm did not benefit from right arm training. However, the left arm did benefit from right arm training for end position error, but not the right arm. These results support the dynamic dominance hypothesis that holds that preferred hemisphere mechanisms underlie specification of the shape and direction of the movement trajectory, while the dominant hemisphere specializes in final limb position (Wang and Sainburg, 2004, 2006). However, the study did not provide information on relative timing so the concept of motor equivalency could not be evaluated completely. One study that was somewhat more relevant for evaluating motor equivalency was performed by Pan and van Gemmert (2013). They had right-handed participants make movements on a digitizing tablet to four targets in four directions beginning with their left or right hands. After practice under normal visual feedback conditions, the feedback was rotated 45^°^ and practice was provided either the left or the right hand. As in the Sainburg and Wang’s (2002) study, asymmetric transfer was shown. Practicing with the right hand under rotated visual feedback conditions showed positive transfer to the unpracticed left hand as reduced movement time, trajectory length, normalized jerk, and initial direction error. The ratio of the primary submovement to the total time and the length from the primary submovement to the target also showed transfer effects. However, practice with the left hand under rotated feedback conditions only showed transfer to the right hand for movement time, trajectory length, and normalized jerk. Apparently, the relative timing pattern learned by the right hand was utilized by the left hand, supporting the concept of motor equivalency, but not for the opposite direction.

## MOTOR EQUIVALENCY IN BIMANUAL MOVEMENTS

Next, we turn to the work on simultaneous bimanual movements when both limbs make aiming movements to either the same or different targets at the same time. In their first experiment, Kelso et al. (1983) kinematic analyses of bimanual aiming movements using Fitts’ (1954) task where participants made movements to combinations of easy (target width, *W* = 7.2 cm, distance, *A* = 6 cm, ID = 0.74) and hard (*W* = 3.6 cm, *A* = 24 cm, ID = 3.74) targets. When both hands moved to similar targets, the average interlimb difference in movement time was 6 ms. When the hands moved to targets of different difficulty the average movement time difference was 23 ms. Regardless of the bimanual condition (easy–easy, hard–hard, easy–hard) the time of peak velocity and the time of maximum vertical displacement were very similar for both limbs. Although the relative timing of the kinematic patterns was not provided, when referring to the easy–hard movement condition they reported, “although the paths of the two trajectories are obviously different, their form looks remarkably alike as if one were an expanded (or contracted) version of the other.” Their second experiment also supported the notion that both limbs were controlled by the same motor program when a hurdle was placed in the path of one of the hands as both hands moved to hard targets. Most participants showed spatially symmetrical movements in both hands even though only one hand was required to clear the hurdle, while three subjects showed relatively independent movements in each hand. Fowler et al. (1991) replicated Kelso et al.’s (1983) work and added a more difficult target condition (*W* = 2 cm, *A* = 36 cm, ID = 5.17). When the 0.74 ID and 3.74 ID movements were combined the movement time differences were 33 ms, and 57 ms when the 0.74 ID and 5.17 ID were combined. Analysis of the resultant velocity and acceleration indicated that participants were very consistent within and between testing conditions, particularly when moving to the same target in each hand. When different targets were involved, greater between-subject variation was noted. Some participants showed a high degree of synchronization, while others showed a lack of synchronization. In general, moving to different targets caused interlimb differences in the form of the velocity-time curve depending on which hand moved to the harder target. However, the relative timing of the various peaks in velocity and acceleration were not computed so the implication for motor equivalency could not be determined.

However, Sherwood (1994) examined the relative timing in simultaneous bimanual aiming movements involving the same or different distances in each hand. The right hand goal was always 60^°^, and the left hand moved either 30^°^, 40^°^, 50^°^, or 60^°^. As expected, the left hand overshot the 30^°^ and 40^°^ targets and the right hand undershot the 60^°^ target showing assimilation effects. However, an analysis of the relative timing of three landmarks (time of peak positive velocity, the time of the intermediate zero crossing, and the time of peak negative velocity) were very similar for the left hand (31, 51, and 75%) compared with the right hand (29, 51, and 75%). Clearly over changes in distance the same GMP controlled each hand.

More recently, Maslovat et al. (2008) provided an interesting test of motor equivalence by contrasting bimanual movements initiated by a control tone (82 dB) or a startle tone (124 dB) where the left hand goal was 10^°^ and the right hand goal was 20^°^ of elbow extension. The endpoint error was greater in the left hand compared with the right hand, particularly on the startle trials. As expected the premotor reaction time was reduced by about 50% on the startle trials relative to the control trials. However, the velocity profiles were strongly correlated across limbs (all above 0.90) for both control and startle trials. Also, this study provided an excellent analysis of the electromyographic (EMG) pattern underlying the control and startle trials. They recorded surface EMG from the left and right triceps brachii and biceps brachii, and the left sternocleidomastoid muscles on control and startle trials. On both startle and control trials the expected triphasic EMG pattern was shown in both limbs with a single burst of antagonist activity appearing between the two agonist bursts. The onset and offset times of the agonist and antagonist muscles were invariant across limbs and conditions, strongly supporting the concept of motor equivalency. Interestingly, sternocleidomastoid muscle activity was only shown on the startle trials and its activity preceded the agonist muscle activity by an average of 50 ms.

## SUMMARY: FACTORS INFLUENCING MOTOR EQUIVALENCY

The evidence for motor equivalency presented in the previous sections was clearly mixed with studies showing evidence both for and against the concept. The goal for this section is to identify general factors that influence the presence of motor equivalency. One factor that has a strong influence on motor equivalency is the task involved. Evidence for motor equivalency is strong when one- or two-dimensional movements are made to predicable target locations (Poston et al., 2009; Zuoza et al., 2009). Because of the stable environmental conditions the GMP can be prepared in advance and run without a concern for online corrections. The learned invariant characteristics of the GMP can be easily applied to the both limbs by simply changing the muscle group used for the task. The importance of preprograming was also emphasized by the work of Maslovat et al. (2008) when rapid movements were produced by triphasic EMG patterns of both limbs when activated by startle responses.

The second task type to show strong evidence for motor equivalency was when simultaneous bimanual movements were made to either the same of different targets. According to models of bimanual control (Marteniuk and MacKenzie, 1980; Marteniuk et al., 1984), the same GMP is used to control both limbs, but different parameters could be assigned to each limb if needed. If both limbs need to travel the same distance or move to the same sized targets, the same level of force or amplitude could be applied to both limbs. But, if different distances are required, then different levels of force could be applied to the each limb independently. In both cases, the invariant features of the GMP should be preserved in each limb. The reviewed studies by Sherwood (1994) and Maslovat et al. (2008) clearly support the concept of motor equivalency in bimanual movements.

Mixed support for motor equivalency was shown when movement corrections were required in order to reach the target. Carson et al. (1993) showed that more relative time was required during the decelerative phase compared with the accelerative phase, but this pattern was the same for both preferred and non-preferred limbs. On the other hand, when participants have to adapt to new visual feedback conditions when the target location is rotated, the amount of transfer depends on the order of practice. Pan and van Gemmert (2013) showed that the relative timing pattern learned by the right hand was used by the left hand, but not for the opposite direction. It could be that additional practice was required by the left hand in order to attain motor equivalency.

The least amount of support for the concept of motor equivalency comes from unconstrained tasks like three-dimensional aiming and throwing, but for different reasons. In three-dimensional aiming tasks, the relative timing pattern in deceleration is frequently disrupted by target impact (Todor and Cisneros, 1985), so many practice trials cannot be used for analysis. But, when the relative timing of both accelerative and decelerative phases were available for analysis, the support for consistent relative timing and motor equivalency were shown (Haaland and Harrington, 1989). As for throwing tasks, several studies did not report relative timing measures so the concept of motor equivalency could not be determined (e.g., Hore et al., 1996). However, the Hore et al.’s (2005) study reported some evidence for a consistent relative timing in the preferred limb but not in the non-preferred limb.

A second major factor determining whether support is shown for motor equivalency is the measures that are taken. Motor equivalency is evaluated on the basis on relative time or relative force measures, but several of the studies in this review did not report relative timing measures, but still provided important information on bilateral transfer. For example, Sainburg and Kalakanis (2000) provided an analysis of joint torques and relative joint motions for both the left and right hands instead of relative timing measures. Their work suggested that the limbs were controlled by different GMPs, but without relative timing measures the conclusions were not definitive. If the concept of the GMP is expanded to include measures like relative joint motions then this type of work would be more relevant for the concept of motor equivalency.

Finally, practice likely has an important role in establishing the GMP in the non-preferred limb, particularly in unconstrained throwing tasks. McDonald et al. (1989) provided 1250 practice throws for the non-preferred limb and showed strong correspondence between kinematic patterns of both limbs, suggesting that considerable practice was required before motor equivalency could be attained.

### RECONCILING MOTOR EQUIVALENCY AND ASYMMETRICAL BILATERAL TRANSFER

As noted at the beginning of the paper, one of the challenges for the concept of motor equivalency was the notion that each hemisphere contributes differently to the motor control process. In a majority of the studies reviewed, the accuracy of the preferred and non-preferred limbs were equivalent, supporting Bernstein’s notion that the same motor program could easily be used to control both limbs. However, in most of these studies, errors could only be made in one dimension. In studies where direction error could be dissociated from final position error, it has been shown that the left (preferred) hemisphere provides the shape and the direction of the movement, while the right (non-preferred) hemisphere specializes in final limb position (Sainburg and Wang, 2002; Wang and Sainburg, 2004, 2006). It could be that a GMP is available for the control for either limb, but depending on the task requirements, the program could be adapted to fit the situation. If the GMP can carry out the task without need of movement corrections, then the program can be applied to both limbs expressing motor equivalency. If movement corrections are needed to attain the target, then the right hemisphere can become active to initiate the corrective process that would minimize the influence of the original GMP. Secondly, the GMP could specify the relative timing structure of movements with either limb, but manual asymmetries could emerge due to differences in the parameter specification process undertaken at a lower level of the control system. This notion could account for reduced movement variability and more precise force production in the preferred limb relative to the non-preferred limb (Annett et al., 1979; Peters, 1980).

### LIMITATIONS ON THE WORK IN MOTOR EQUIVALENCY AND FUTURE DIRECTIONS

Even though many studies have been conducted on bilateral transfer and motor equivalency in aiming movements, some limitations in the work should be recognized. For example, the finding supporting the concept of motor equivalency in studies on bilateral transfer crucially depend on how the preferred and non-preferred limbs are compared. In most studies reviewed in this paper, the limbs are compared after significant practice has occurred in both limbs (cf., McDonald et al., 1989). In this situation, it is not surprising that the non-preferred limb mirrors the characteristics of the preferred limb, or vice versa. Perhaps the ultimate test of the concept of motor equivalency would be to examine the invariant characteristics of the non-preferred limb on the initial practice trials following extensive practice with the preferred limb. Future work could establish the amount of transfer in this and other contexts (Robinson et al., 2010).

A second limitation of the current work on motor equivalence is the dependence on mean scores for relative timing and accuracy, for example. In many of the reviewed studies, the relative timing pattern for the preferred and non-preferred limbs were very similar, and not significantly different. However, the analysis of the mean scores ignores individual differences. Strong evidence for motor equivalency should be reflected in positive within-subject correlations in invariant characteristics it addition to similar means. However, such correlations are reported infrequently (McDonald et al., 1989 is an exception) so future work could establish the strength of the coordination between limbs. Also, it is quite likely that some performers would show greater evidence for motor equivalency than others based on factors such as past motoric experience, movement efficiency, genetics, strategies, or in general, intrinsic dynamics (Kelso, 1999). Perhaps future studies could follow the lead of Kelso et al. (1983) and Fowler et al. (1991) whom reported individual differences in bimanual coordination of aiming responses, for example.

Finally, the assumption that movement kinematics are a direct result of the GMP could be called into question. For example, the kinematic pattern of aiming movements, regardless of limb, could be a function of efficiency rather than specified by the program. As accuracy demands increase, performers spend more time in the deceleration phase (Carson et al., 1993) perhaps to use more efficient feedback-based processing than central control. Future studies could evaluate movement efficiency using EMG, for example, to help distinguish between the GMP and efficiency explanations for the kinematic pattern in aiming movements.

### Conflict of Interest Statement

The author declares that the research was conducted in the absence of any commercial or financial relationships that could be construed as a potential conflict of interest.
